# Assessing Pediatric Traumatic Brain Injury and Health-Related Quality of Life in Three Eastern European Countries

**DOI:** 10.1016/j.pedhc.2025.09.022

**Published:** 2025-11-03

**Authors:** Madalina Adina Coman, Diana Dulf, Patricia Maria Marga, Artashes Tadevosyan, Nino Chikhladze, Angela Cazacu-Stratu, Corinne Peek-Asa

**Affiliations:** Assistant professor, Department of Public Health, College of Political, Administrative and Communication Sciences, Babes-Bolyai University, Cluj-Napoca, Romania; Assistant professor, Department of Public Health, College of Political, Administrative and Communication Sciences, Babes-Bolyai University, Cluj-Napoca, Romania; Research assistant, Department of Public Health, College of Political, Administrative and Communication Sciences, Babes-Bolyai University, Cluj-Napoca, Romania - PhD candidate, Department of Public Health, College of Political, Administrative and Communication Sciences, Babes-Bolyai University, Cluj-Napoca, Romania; Professor, Yerevan State Medical University after Mkhitar Heratsi, Department of Public Health and Health Care - Head of the Department of Public Health and Health Care Organization, Yerevan State Medical University after Mkhitar Heratsi, Department of Public Health and Health Care; Professor, Faculty of Medicine, Department of Public Health, Ivane Javakhishvili Tbilisi State University, Georgia - Head of the Quality Assurance Service at the Faculty of Medicine, Ivane Javakhishvili Tbilisi State University, Georgia; Associate professor, Department of Preventive Medicine, “Nicolae Testemitanu” State University of Medicine and Pharmacy of the Republic of Moldova - Vice-Dean of the Faculty of Medicine, Program study Public Health, “Nicolae Testemitanu” State University of Medicine and Pharmacy of the Republic of Moldova; Office of Research Affairs, Vice Chancellor for Research and Innovation, University of California, San Diego, La Jolla, CA, United States

**Keywords:** Quality of life, brain injuries, traumatic, registries, developing countries

## Abstract

**Introduction:**

Pediatric traumatic brain injury (TBI) is a major global cause of disability, yet its impact in low- and middle-income countries (LMICs) is poorly understood. This study assesses TBI incidence and health-related quality of life (HRQoL) among children in Armenia, Georgia, and Moldova.

**Methods:**

This cross-sectional study used a standardized pediatric TBI registry and the EQ-5D-Y-3L instrument (in Georgia and Moldova). A total of 745 pediatric TBI cases were recorded, with 273 children aged 4–18 completing the HRQoL assessment.

**Results:**

Most TBIs were mild (93.7%). Despite mild severity, 30.6% reported pain/discomfort and 23.8% reported difficulty with usual activities. Older age was significantly associated with lower EQ-VAS scores (*p* = .007).

**Conclusions:**

Even mild pediatric TBIs can negatively affect HRQoL, particularly in older children. Incorporating HRQoL measures into TBI registries in LMICs can support evidence-based care, guide targeted interventions, and inform health policy in resource-limited settings.

## INTRODUCTION

Traumatic brain injury (TBI) is a leading global cause of injury-related death and disability in children and adolescents (Zhong et al., 2025). The World Health Organization predicts that the TBI burden will rise further in the coming years, particularly in low- and middle-income countries (LMICs) where brain injury rates are roughly three times those of high-income nations (Dulf et al., 2021). Childhood TBI accounts for significant mortality as well as potentially prolonged morbidity. Although most pediatric TBIs are mild, with a Glasgow Coma Scale (GCS) between 13 to 15, a significant proportion of even mild injuries can disrupt learning, social engagement and result in long-term symptoms and deficits in multiple domains of health-related quality of life (HRQoL). These deficits span across physical, emotional, social, and school functioning, with little improvement over time (Keenan et al., 2023; Kyösti et al., 2024; Rivara et al., 2011).

Eastern and Central Europe exhibit some of the highest age-standardized TBI prevalence and disability rates worldwide (Zhong et al., 2025). In the Republic of Armenia, Georgia, and the Republic of Moldova, three neighboring LMICs, pediatric TBI is starting to be increasingly recognized as a public health concern, although comprehensive data are limited (Chikhladze et al., 2020; Cociu et al., 2022; Peek-Asa et al., 2022; Sargsyan, 2023). Despite its importance, HRQoL after pediatric TBI has not been studied in these three countries; nearly all work being done in these countries has focused on epidemiology or acute care capacity (Burkadze et al., 2021; Chikhladze et al., 2022; Cociu et al., 2023; Dulf et al., 2021; Peek-Asa et al., 2022). While pediatric TBI appears common in this region, the impact of these injuries on children’s daily functioning and quality of life remains unknown. Therefore, this study addresses the following research question: What are the incidence patterns and HRQoL outcomes of pediatric TBI patients from the Republic of Armenia, Georgia and the Republic of Moldova?

## METHODS

### Study Setting and Study Population

This study was part of the INITIatE project (International Collaboration to Increase Traumatic Brain Injury Surveillance in Europe), supported by the NIH/NINDS [R21NS098850]. It employed a prospective observational design across hospitals in the Republic of Armenia, Georgia, and the Republic of Moldova, with the aim of collecting standardized registry data on TBI cases and associated HRQoL outcomes.

Pediatric traumatic brain injury cases were identified between March and September 2019 in three different pediatric hospitals, one from each country: the Republic of Armenia (“Surb Astvatsamair” Medical Center), Georgia (M. Iashvili Children’s Central Hospital), and the Republic of Moldova (Municipal Children Clinical Hospital, “Valentin Ignatenco”). Pediatric patients (ages 0–18) admitted with a clinical diagnosis of traumatic brain injury (ICD-10: S00–S09.0) were eligible for inclusion in the registry. For the HRQoL component, only children aged 4–18 were included, in line with EQ-5D-Y-3L instrument validation. Children aged 4–7 completed the instrument through proxy reporting by parents or caregivers. The hospitals were chosen based on the high number of pediatric trauma cases they handle. The project was approved by the Ethics Review Boards of each of the partner institutions and participating hospitals before its start.

The data collection had two phases: (1) data were extracted from medical records using the TBI registries and included all pediatric patients admitted to the hospital with a TBI diagnosis, and (2) pediatric patients or their proxy were invited to complete the EQ-5D-Y-3L scale at discharge to document self-reported health outcomes and HRQoL. Trained hospital personnel enrolled eligible study participants, and all patients who agreed with the study protocol signed the informed consent. Information was first recorded on paper forms and then securely entered into REDCap, a web-based data capture system. Monthly data quality checks were conducted to maintain data integrity and completeness. A final number of 283 pediatric patients had a valid EQ-5D-Y-3L completed, with a response rate of 47.2%.

### Study Measures

#### TBI registry

The study used a comparable TBI registry implemented in the three participating countries, which contained core TBI information adapted from existing TBI templates from the EU and NIH and tailored to fit the characteristics of all the countries included in the study, as this was the first pediatric TBI registry implemented in these countries (Burkadze et al., 2021; 2020; Peek-Asa et al., 2022). The variables and codebook used in this study were chosen through an iterative process undertaken by the research team as a part of a larger initiative to enhance TBI surveillance in Eastern Europe. Sample registry templates were initially identified through a literature review and consultations with investigators from NIH and EU-funded registry projects. Based on these, a core set of variables and a codebook were created. These tools were pilot tested using medical records from each of the participating hospitals. However, because medical records were not enough in terms of detail, the research was adapted to a prospective data collection model (Burkadze et al., 2021; Peek-Asa et al., 2022). Variables from the TBI registry explored in the present study include socio-demographic characteristics (age, sex, area where injury occurred, injury intent, type of addressing to the hospital, work-related injury, and place of occurrence of injury) and TBI-related symptoms (GCS score, loss of consciousness, post-traumatic amnesia, consciousness alteration, discharge disposition).

#### HRQoL measures - EQ-5D-Y-3L (formerly known as EQ-5D-Y)

The EQ-5D-Y-3L (EuroQol Research Foundation, 2024) scale in Georgian and Romanian (Moldova’s official language) was completed by self-reporting or proxy reporting (caregivers of patients unable to complete the survey themselves or patients too severely injured to respond on their own) before the patients were discharged from the hospital using country-specific language versions. No EQ-5D-Y-3L data were collected in the pediatric hospital from the Republic of Armenia, as at the time of data collection, the standardized tool was not available in the Armenian language.

EQ-5D-Y-3L consists of a two-part survey: the descriptive system and the visual analog scale (EQ-VAS). The descriptive system intended to measure HRQoL across five dimensions: mobility (walking about), self-care (looking after myself), usual activities (for example, going to school, hobbies, sports, playing, doing things with family or friends), having pain or discomfort, and anxiety/depression (feeling worried, sad or unhappy). Each dimension was rated by each participant (or caregiver) using a three-response option ranging from “1 - no problems” to “3 - a lot of problems”. The EQ-VAS is comprised of a score from 0 (the worst health condition) to 100 (the best health condition) they had at discharge. The EQ-5D-Y-3L has recommendations on how to apply the tool for children and adolescents based on different age ranges, as follows: for children under 3 years old there are no versions of the instrument available; and for children aged 4–7 years a proxy version of the instruments should be used and completed by a parent, caregiver or health professional, for children 8–18 a self-complete version is recommended for this age group (EuroQol Research Foundation, 2024).

### Statistical Analysis

The collected data was cleaned, checked, and the final data-set was exported in SPSS for analysis. Data for socio-demographics, TBI registry variables, and EQ-5D-Y-3L tool were examined. Frequency statistics were computed for the main demographics and TBI-related variables. Relationships between age, sex, GCS score, injury mechanism, and HRQoL were explored using a multiple linear regression model to identify potential associations.

## RESULTS

### Socio-Demographics Characteristics

**FIGURE 1.**
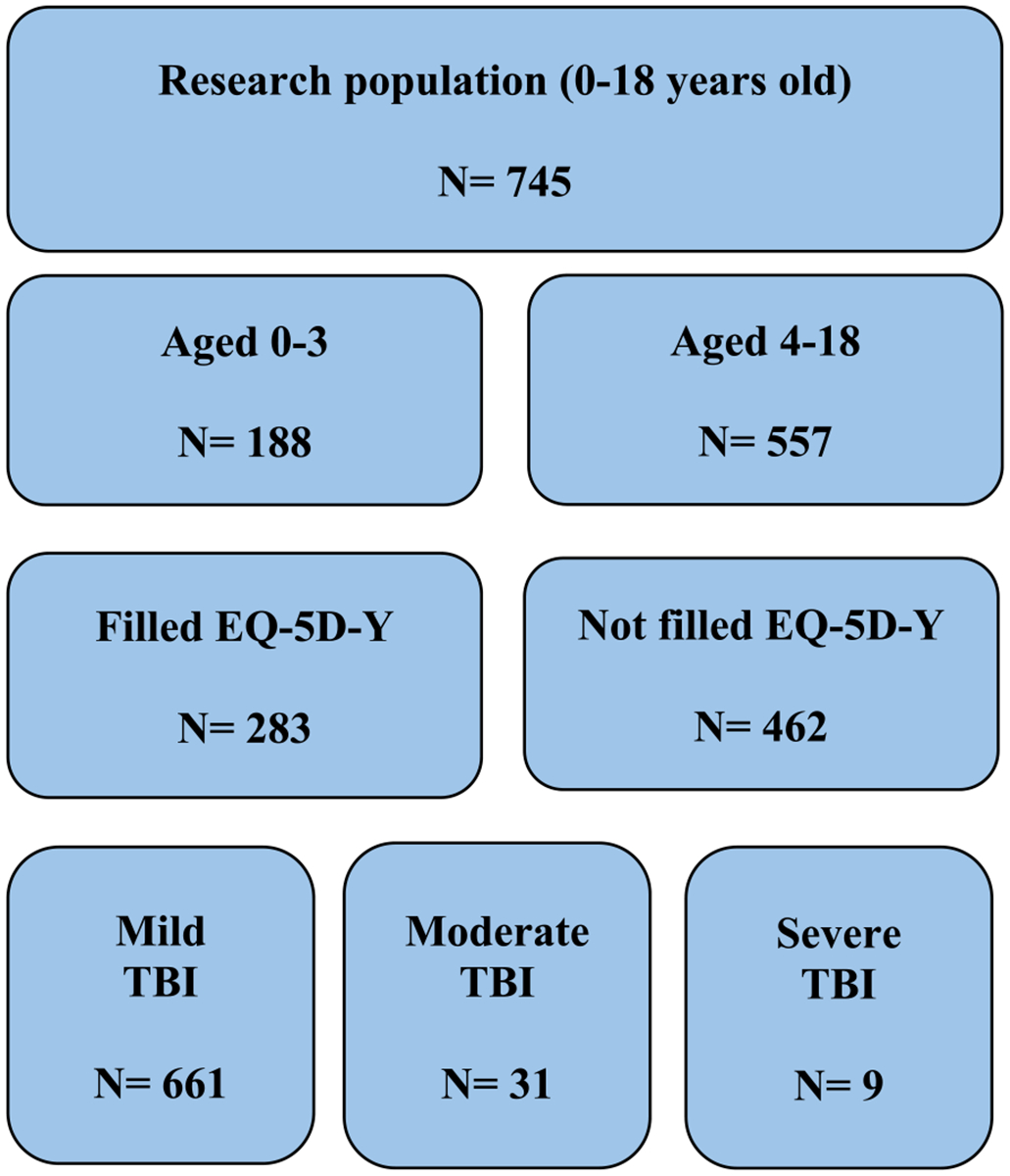
Flowchart of data collection.

Figure 1 shows that the TBI registry in all three countries included patient records of 745 children aged 0–18 with a TBI diagnosis who attended one of the participating hospitals. Out of the entire sample, 188 children aged 0–3 were excluded from the analysis because the EQ-5D-Y-3L, instrument is not validated for children under 4. Out of the remaining 557 cases, 273 completed the HRQoL assessment. Of these total cases, 110 (19.7%) were from the Republic of Armenia, 296 (53.1%) from Georgia, and 151 (27.1%) from the Republic of Moldova, reflecting the relative size of the hospitals in each country and their admitted number of patients and registering a participation rate of 47.2%.

In each hospital sample, most of the injuries were unintentional (93.5%) and most injuries occurred in urban areas (86%) (Table 1). The main injury mechanism was falls in the Republic of Moldova (55.6%) and in Georgia (52.7%), while in Armenia, the most frequent injury mechanism was struck by or against (38.9%).

### TBI-Related Symptoms of the Sample

Most patients included in the TBI registry suffered a mild TBI (N=504), with the Republic of Moldova having the most patients with moderate TBI (14.8%). In Armenia, all cases except three were mild TBI (Table 2).

### HRQoL of Pediatric Patients Treated in Three Large Hospitals

The study sample for HRQoL assessment consisted of 273 children between the ages of 4 and 18 years in the Republic of Moldova and Georgia. While the proportion of respondents reporting issues in the mobility (9.5%), self-care (11.4%), and anxiety/depression (14.7%) dimensions were relatively low, a higher proportion of the participants experienced problems in their usual activities (23.8%) and pain/discomfort (30.6%) (Table 3). The EQ-VAS ranged between 0 (worst health they can imagine) to 100 (best health they can imagine), with a mean of 92 (Table 3).

### Linear Regression (EQ-VAS, GCS, Injury Mechanism, Age and Sex)

A multiple linear regression model was conducted to examine whether age, sex, injury mechanism, and GCS severity predict EQ-VAS scores. The overall model was statistically significant (*p*-value = .033) and explained 3.8% of the variance in EQ-VAS score (R^2^= 0.038). Among the predictors, only age was statistically significant (***β***= −0.470, *p*-value = .007), indicating that older participants tend to report lower EQ-VAS scores. GCS severity, injury mechanism, and sex were not statistically significant predictors (Table 4).

## DISCUSSION

To our knowledge, this is the first study to examine the HRQoL following pediatric TBI in the Republic of Armenia, Georgia, and the Republic of Moldova.

The results of our study population are consistent with other published results in terms of higher TBI risk for younger boys and mode of transportation to the hospital (Allen et al., 2023; Dewan et al., 2016; Dulf et al., 2021; Peek-Asa et al., 2022). In our study, a small percentage of youth experienced moderate to severe GCS scores, roughly 6% of the sample. Even if the majority of patients sustained mild TBIs and reported some problems on the HRQoL tool, especially for pain/discomfort and usual activities, literature shows that even mild TBIs are associated with long-lasting conditions and hinder patients’ quality of life, especially for children (Fineblit et al., 2016; Tunthanathip et al., 2021; Vu et al., 2019).

Almost 15% of participants reported symptoms of anxiety/depression after TBI, which is consistent with previous studies that show that 11 to 45% of children have an increased risk of different mental health conditions and lower HRQoL following a TBI (Bradbury et al., 2021). Existing research in the field shows that poorer HRQoL is linked with more severe depressive symptoms, many years post-TBI, reduced cognitive flexibility, and perceived social communication difficulty (Krasny-Pacini et al., 2017; Li & Liu, 2013; Ryan et al., 2013, 2019). These findings point to the necessity of improved screening and therapeutic strategies to address emotional difficulties in children with TBI. By focusing on modifiable risk factors such as anxiety and depression, early interventions can play a critical role in facilitating recovery and enhancing overall well-being after a TBI diagnosis.

Apart from emotional problems, almost 24% of our respondents reported having difficulties with their usual daily activities, an aspect that is often associated with TBI. However, studies conducted in the field of pediatric TBI show that by young adulthood, children who experienced a TBI report a significantly worse HRQoL for the physical domain, as residual physical and functional disabilities are common experiences in the long term after pediatric TBI (Fuentes et al., 2018; Ryan et al., 2019; Zonfrillo et al., 2013). These results highlight the fact that TBI isn’t just an immediate medical concern, but has lasting consequences that can affect independence, mobility, and overall well-being. Understanding this helps in developing better rehabilitation strategies and support systems for affected individuals (Ryan et al., 2019). In most cases, children are released from the hospital without specific follow-up protocols, and follow-up for post-TBI symptoms could be explored to reduce the impact of post-TBI symptoms and impacts.

Moreover, the scarcity of TBI data in these three countries and valid estimates of the burden of TBI in children pose a limited capacity to quantify the HRQoL for these types of patients. Also, existing instruments for measuring HRQoL for pediatric TBI at the point of data collection were mostly generic instruments, and not focused on disease-specific HRQoL, which are proven to show more sensitivity to the consequences of a specific health condition, such as TBI (Von Steinbuechel et al., 2023). Our results can also be explained by the fact that data were collected using a generic HRQoL tool and not a TBI-specific patient-reported outcome measure (PROM), which did not exist at the point of data collection. Using TBI PROM measures could help improve the quality of data collected by focusing specifically on the experiences and difficulties associated with the TBI-specific symptoms, and focusing on collecting data more accurately for different age groups (Zeldovich et al., 2024).

Results from the regression model indicated an association between age and HRQoL, suggesting that older children reported slightly lower health-related quality of life following a TBI. No significant association was found between sex, GCS, injury mechanism and HRQoL in this sample. These findings align with previous research showing that HRQoL tends to decrease with age in pediatric populations, as adolescents often report more subjective health complaints than younger children (Haraldstad et al., 2023; Wang et al., 2022). One explanation for the results might be the fact that 34.6% of the sample was between 4–7 years old, and parents or another caregiver might have acted as a proxy for them, interfering with the completion of the HRQoL tool, as a systematic review evaluating the EQ-5D-Y already demonstrated it (Noyes & Edwards, 2011). Another explanation might reside in the findings of the literature showing that subjective health complaints are reported to increase from childhood to adolescence after TBI; hence, younger patients usually report a better HRQoL compared to their older counterparts (Fineblit et al., 2016).

Our study has some limitations. First, no pediatric data for HRQoL were present for the Republic of Armenia, because the tool was not available in armenian. However, our study does provide valuable information on pediatric TBIs, partially addressing the lack of such data in this country. Moreover, we used prospective data collected at one point in time with a TBI registry and no follow-up points of data collection were used for this study. As for most studies using the same study design, the sample comprised pediatric patients having uncomplicated TBI, hence the lack of variability in the sample. However, having data collected systematically with a tool such as a TBI registry will offer longitudinal data and better evidence of identifying common causes of pediatric TBI, improving care, guiding policymaking decisions, and advancing scientific understanding of connection between TBI and physical and emotional HRQoL for youth.

Better understanding of the HRQoL of pediatric patients should be a focus area, especially in LMIC countries, where data is scarce in the pediatric TBI area. Integrating HRQoL measures in TBI Registries allows for a more patient-centered approach, capturing the functional, emotional, and social consequences of TBI. This is particularly important in LMICs like the Republic of Armenia, Georgia, and the Republic of Moldova, where such data is scarce and healthcare resources may be limited. Further research should focus on developing and using TBI registries to better understand the reality in the TBI pediatric population by using age-appropriate TBI-specific PROM tools to measure the HRQoL for youth who experienced TBI over longer periods to observe differences in time. Moreover, other variables that influence HRQoL should be observed in the future, such as socio-economic status, social support and include more severe cases as well, as they prove to impact the perceived HRQoL of pediatric patients (Dipnall et al., 2021; Ryder et al., 2020).

Given the scarcity of such data in these regions, this study contributes with valuable insights that can inform healthcare policies, resource allocation, and the development of targeted interventions to improve the long-term outcomes of children affected by TBI. Furthermore, the use of a TBI Registry enhances the reliability and comprehensiveness of the findings, supporting evidence-based decision-making in these underrepresented settings.

## Figures and Tables

**TABLE 1.** Socio-demographic characteristics of pediatric hospital-admitted patients with a TBI

	Moldova		Armenia		Georgia		All	
Characteristics	N	%	N	%	N	%	N	%
**Age**								
4–7	58	38.4	44	40	91	30.7	193	34.6
8–11	34	22.5	34	30.9	107	36.1	175	34.1
12–15	43	28.5	22	20	70	23.6	135	24.2
16–18	16	10.6	10	9.1	28	9.5	54	9.7
**Total**	151	100	110	100	296	100	557	100
**Mean age**	9.78		9.29		9.87		9.73	
**Sex**								
Male	98	64.9	71	64.5	187	63.2	356	63.9
Female	53	35.1	39	35.5	109	36.8	201	36.1
**Total**	151	100	110	100	296	100	557	100
**Area where injury occurred**								
Urban area	146	96.7	80	73.4	252	85.1	478	86
Rural area	5	3.3	27	24.8	43	14.5	75	13.5
Unknown	0	0	2	1.8	1	0.3	3	0.5
**Total**	151	100	109	100	296	100	556	100
**Injury intent**								
Unintentional	131	86.8	104	95.4	285	96.3	520	93.5
Intentional self-harm	1	0.7	0	0	0	0	1	0.2
Assault (violence)	19	12.6	3	2.8	8	2.7	30	5.4
Other/unknown	0	0	2	1.8	3	1	5	0.9
**Total**	151	100	109	100	296	100	556	100
**Work-related injury**								
Yes	15	9.9	0	0	1	0.3	16	2.9
No	136	90.1	109	100	295	99.7	540	97.1
**Total**	151	100	109	100	296	100	556	100
**Injury mechanism**								
Road traffic injury	47	31.1	34	31.5	36	12.2	117	21.1
Fall	84	55.6	14	13	156	52.7	254	45.8
Assault (violence)	19	12.6	3	2.8	8	2.7	30	5.4
Struck by/or against	1	0.7	42	38.9	96	32.4	139	25
Other	0	0	15	13.9	0	0	15	2.7
**Total**	151	100	108	100	296	100	555	100
**Type of arrival**								
Walk-in	0	0	57	52.3	2	0.7	59	10.6
Ambulance	151	100	48	44	196	66.2	395	71
Private/public vehicle	0	0	2	1.8	96	32.4	98	17.6
Other	0	0	2	1.8	2	0.7	4	0.7
**Total**	151	100	109	100	296	100	556	100
**Place of occurrence**								
Home	33	21.9	48	43.6	71	24	152	27.3
Institutions	47	31.1	2	1.8	86	29.1	135	24.2
Sports and recreational areas	18	11.9	12	10.9	88	29.7	118	21.2
Streets and highways	38	25.2	39	35.5	40	13.5	117	21
Commercial areas	10	6.6	1	0.9	1	0.3	12	2.2
Countryside	5	3.3	3	2.7	3	1	11	2
Other/unspecified	0	0	5	4.5	7	2.4	12	2.2
**Total**	151	100	110	100	296	100	557	100

**TABLE 2.** TBI-related symptoms from the Republic of Armenia, Georgia, and the Republic of Moldova

	Moldova		Armenia		Georgia		All	
Characteristics	N	%	N	%	N	%	N	%
**GSC Score**								
3–8 (severe)	6	4	1	1.1	2	0.7	9	1.7
9–12 (moderate)	22	14.8	3	3.2	0	0	25	4.6
13–15 (mild)	121	81.2	89	95.7	294	99.3	504	93.7
**Total**	149	100	93	100	296	100	538	100
**Loss of consciousness**								
Yes	48	31.8	46	42.2	103	34.8	197	35.4
No	72	47.7	58	53.2	186	62.8	316	56.8
Suspected	31	20.5	1	0.9	5	1.7	37	6.7
Unknown	0	0	4	3.7	2	0.7	6	1.1
**Total**	151	100	109	100	296	100	556	100
**Post-traumatic amnesia**								
Yes	33	21.9	47	41.2	43	14.5	123	22.1
No	73	48.3	59	54.1	253	85.5	385	69.2
Suspected	45	29.8	2	1.8	0	0	47	8.5
Unknown	0	0	1	0.9	0	0	1	0.2
**Total**	151	100	109	100	296	100	556	100
**Consciousness alteration**								
Yes	27	17.9	19	17.6	3	1	49	_8.8_
No	49	32.5	87	80.6	269	90.9	405	73
Suspected	75	49.7	1	0.9	1	0.3	77	13.9
Unknown	0	0	1	0.9	23	7.8	24	4.3
**Total**	151	100	108	100	296	100	555	100
**Headache**								
Yes	151	100	95	88	290	98.3	536	96.8
No	0	0	13	12	5	1.7	18	3.2
**Total**	151	100	108	100	295	100	554	100
**Nausea**								
Yes	143	94.7	83	77.6	237	80.3	463	83.7
No	8	5.3	24	22.4	58	19.7	90	16.3
**Total**	151	100	107	100	295	100	553	100
**Vomiting**								
Yes	139	92.1	72	67.3	89	30.2	300	54.2
No	12	7.9	35	32.7	206	69.8	253	45.8
**Total**	151	100	107	100	295	100	553	100
**Balance**								
Yes	82	54.3	10	9.3	19	6.4	111	20.1
No	69	45.7	97	90.7	276	93.6	442	79.9
**Total**	151	100	107	100	295	100	553	100
**Dizziness**								
Yes	33	21.9	57	43.8	235	79.7	325	58.9
No	118	78.1	49	46.2	60	20.3	227	41.1
**Total**	151	100	106	100	295	100	552	100
**Visual**								
Yes	20	13.2	3	2.8	11	3.7	34	6.1
No	131	86.8	104	97.2	284	96.3	519	93.9
**Total**	151	100	107	100	295	100	553	100
**Fatigue**								
Yes	81	53.6	32	20.2	225	76.3	338	61.2
No	70	46.4	74	69.8	70	23.7	214	38.8
**Total**	151	100	106	100	295	100	552	100
**Light**								
Yes	80	53	13	12.1	2	0.7	95	17.2
No	71	47	94	87.9	293	99.3	458	82.8
**Total**	151	100	107	100	295	100	553	100
**Fracture of the skull**								
Yes	111	73.5	37	33.6	30	10.1	178	32
No	40	26.5	73	66.4	265	89.5	378	67.9
Unknown	0	0	0	0	1	0.3	1	0.2
**Total**	151	100	110	100	296	100	557	100
**Concussion**								
Yes	143	94.7	67	60.9	273	92.2	483	86.7
No	8	5.3	43	39.1	23	7.8	74	13.3
**Total**	151	100	110	100	296	100	557	100
**Discharge Disposition**								
Home	118	78.1	108	98.2	289	97.6	515	92.5
Rehab	25	16.6	0	0	0	0	25	4.5
Nursing home/long-term care	0	0	0	0	1	0.3	1	0.2
Acute care	8	5.3	0	0	1	0.3	9	1.6
Unknown	0	0	2	1.8	5	1.7	7	1.3
**Total**	151	100	110	100	296	100	557	100

**TABLE 3.** EQ-5D-Y-3L dimensions in Georgia and the Republic of Moldova

	Moldova		Georgia		All	
EQ5D DIMENSIONS	N	%	N	%	N	%
**Mobility (walking around)**						
I have no problems walking around	114	95.8	133	86.4	247	90.5
I have some problems walking around	5	4.2	18	11.7	23	8.4
I have a lot of problems walking around	0	0	3	1.9	3	1.1
**Total**	119	100	154	100	273	100
**Self-care (looking after myself)**						
I have no problems taking a bath or shower by myself or getting dressed by myself	106	89.1	136	88.3	242	88.6
I have some problems taking a bath or shower by myself or getting dressed by myself	13	10.9	14	9.1	27	9.9
I have a lot of problems taking a bath or shower by myself or getting dressed by myself	0	0	4	2.6	4	1.5
**Total**	119	100	154	100	273	100
**Usual activities (for example, going to school, hobbies, sports, playing, doing things with family or friends)**						
I have no problems doing my usual activities	99	83.2	109	70.8	208	76.2
I have some problems doing my usual activities	20	16.8	42	27.3	62	22.7
I have a lot of problems doing my usual activities	0	0	3	1.9	3	1.1
**Total**	119	100	154	100	273	100
**Pain/discomfort (having pain or discomfort)**						
I have no pain or discomfort	93	78.2	96	62.3	189	69.2
I have some pain or discomfort	26	21.8	50	32.5	76	27.8
I have a lot of pain or discomfort	0	0	8	5.2	8	2.8
**Total**	119	100	154	100	273	100
**Anxiety/depression (feeling worried, sad or unhappy)**						
I am not worried, sad, or unhappy	100	84	133	86.4	233	85.3
I am a little worried, sad, or unhappy	19	16	20	13	39	14.3
I am very worried, sad, or unhappy	0	0	1	0.6	1	0.4
**Total**	119	100	154	100	273	100
	**Mean**	**Median**	**Mean**	**Median**	**Mean**	**Median**
**EQ-VAS**	92.65	92	91.87	100	92.21	95

**TABLE 4.** Linear regression (EQ-VAS, GCS, injury mechanism, age and sex)

Variable	B	95% CI		β	t	*p*
		Lower	Upper			
**Sex**	−1.400	−4.048	1.247	−0.064	−1.041	.299
**Age**	−0.470	−0.809	−0.131	−0.170	−2.732	.007
**Injury mechanism**	−0.167	−1.396	1.062	−0.016	−0.268	.789
**GCS**	−2.174	−7.307	2.960	−0.52	−0.834	.405
